# Comparative Analysis of the Nutritional Components and Antioxidant Activities of Different *Brassica juncea* Cultivars

**DOI:** 10.3390/foods9060840

**Published:** 2020-06-26

**Authors:** Hee-Yeon Kwon, Sun-Il Choi, Hye-In Park, Seung-Hyun Choi, Wan-Sup Sim, Jin-Hui Yeo, Ju-Hyun Cho, Ok-Hwan Lee

**Affiliations:** 1 Department of Food Science and Biotechnology, Kangwon National University, Chuncheon 24341, Korea; sosakwon1@naver.com (H.-Y.K.); docgotack89@hanmail.net (S.-I.C.); dls9615@naver.com (H.-I.P.); zzaoszz@naver.com (S.-H.C.); simws9197@naver.com (W.-S.S.); 2 Jeongseon Agriculture Technology & Extension Center, Jeongseon 26103, Korea; 1257@korea.kr; 3 Haram Central Research Institute, Cheongju 28160, Korea

**Keywords:** *Brassica juncea*, antioxidant, nutritional components, comparative analysis, different cultivars

## Abstract

The purpose of this study was to compare the nutritional components and antioxidant activities of two different cultivars of *Brassica juncea* (Dolsan, Yeosu, Korea (BJD) and (Jeongseon, Gangwon, Korea (BJJ)). We investigated the proximate composition (moisture, crude ash, crude protein and crude lipid), antioxidant activities (2,2-Diphenyl-2-picrylhydrazil (DPPH) scavenging activity and ferric reducing antioxidant power (FRAP)), total phenol content, total flavonoid content and sinigrin content by high-performance liquid chromatography analysis. Our results show that the proximate compositions of BJD and BJJ were not significantly different. However, both the DPPH radical scavenging and FRAP activities of the BJJ extracts were higher than those of the BJD extracts. The total phenol contents of the BJD and BJJ extracts were 6.56 and 9.80 mg gallic acid equivalent/g, respectively. The total flavonoids content of the BJD and BJJ extracts were 20.92 and 34.81 mg rutin equivalent/g, respectively, whereas the sinigrin contents, one of the major compounds in BJD and BJJ extracts, were 16.16 mg/g and 11.73 mg/g, respectively. In this study, we confirmed that, by comparing BJJ and BJD, the sinigrin content of BJD was higher than that of BJJ, but the antioxidant activity and phenol content of BJD were superior to that of BJJ.

## 1. Introduction

The leaf mustard *Brassica juncea* is one of the vegetables belonging to the Brassicaceae family. It originated from China but is widely cultivated in Korea and Japan. It is a biennial plant mainly used for its edible leaves, stems and seeds to make mustard [1]. *B. juncea* has excellent aroma and is widely grown as a main ingredient of kimchi in Korea [2]. The unique and the pungent flavor of *B. juncea* is mainly caused by allyl isothiocyanate, a major volatile and spicy ingredient in mustard. The pungent taste is generated when the enzyme myrosinase degrades sinigrin, the glucosinolate present in *B. juncea*, to release allyl isothiocyanate [3]. *B. juncea* is rich in secondary metabolites, such as flavonoids, polyphenols, and sulfur compounds [4].

The phenolic hydroxyl (−OH) group present in the phenolic compounds has the property of binding to proteins and is known to have physiological activities, such as antioxidant, anticancer and antimicrobial effects [5]. The flavonoids are present in almost all parts of the plant, such as the leaves, flowers, fruits, stems and roots, and are reported to have antioxidant properties that can help remove reactive oxygen species (ROS), as they are effective as antiviral, anti-inflammatory and anti-cancer agents [6,7]. These substances show beneficial health-related effects in humans by enhancing the immune system, contributing to the maintenance of health, preventing cancer, and they also have ROS inhibitory capability with high antioxidant activity [5,8]. The ROS generated in the body during intracellular metabolism are involved in various biological processes, such as cell differentiation and DNA expression [9,10]. Therefore, maintaining the homeostasis of ROS is crucial for cell growth and survival. This imbalance of ROS can also damage the DNA, causing mutations or cancers [11]. Many human diseases, such as arteriosclerosis, diabetes, stroke, hepatitis, myocardial infarction, nephritis, atopic dermatitis and Parkinson’s disease, are also associated with ROS generation [12,13].

The damage that is caused by ROS generation can be prevented to a certain degree by the internal defense mechanism that use enzymes, such as superoxide dismutase, catalase and antioxidants [14]. However, due to various environmental factors, such as environmental hormones, alcohol, smoking and the weakening of defense mechanisms, the amount of active oxygen in the body can greatly increase, and it may not be able to protect itself from the ensuing damage. Therefore, studies investigating the development of antioxidants to inhibit the generation of active oxygen have increased. As a result, synthetic antioxidants, such as butylated hydroxyanisole and butylated hydroxytoluene, with strong antioxidant capabilities have been developed, but the side effects, such as hepatotoxicity, have also raised concerns [15]. Therefore, research on the development of natural antioxidants using safe natural materials is being actively pursued [16].

*B. juncea* is cultivated in Dolsan, Yeosu, Korea. Since Yeosu is located in the middle of the south coast of the Korean peninsula, the cultivation site has a characteristic coastal climate. In recent years, *B. juncea* has been selected as a special agricultural crop in Jeongseon, Gangwon-do, Korea, and is actively grown there. Since Jeongseon is a mountainous region, the cultivation of Jeongseon is characterized by high altitude and climate in mountainous areas. Studies on *B. juncea* in Korea, such as those involving component analysis and antibacterial activity, have been conducted only on *B. juncea* cultivated in Dolsan (BJD) [17,18]. However, there have been no studies on *B. juncea* cultivated in Jeongseon (BJJ). Therefore, this study aims to establish the research data on BJJ that has not been studied by comparing the food composition of BJD and BJJ according to the cultivation characteristics of the Dolsan province in Yeosu, Jeollanam-do and Jeongseon, Gangwon-do.

## 2. Materials and Methods

### 2.1. Materials

The BJD used in this study was purchased from Food Story (Dolsan, Yeosu, Jeollanam-do, Korea) and the BJJ was obtained from Jeongseon Agricultural Technology Center (Jeongseon, Gangwon-do, Korea). Both crops used in the experiment were cultivated for one month in October 2017, and the average temperature, rainfall and sunshine for the month are shown in Table 1 [19]. In the experiment, mainly intake leaves and stems were used. The raw materials used in the experiment were washed thoroughly to remove foreign substances, then grounded and homogenized into powder that was used for the proximate analysis. In order to make extracts for the analysis of antioxidant activity, 20 g of pulverized *B. juncea* was added to 400 mL of 80% ethanol and then extracted at 70 °C for 2 h. The extracts of BJD and BJJ were diluted to 0.5, 1.0, and 5.0 mg/mL concentrations for antioxidant experiments, and 10 mg/mL for sinigrin analysis. Sinigrin, 2,2-Diphenyl-1-picrylhydrazyl (DPPH), 2,4,6-Tripyridyl-s-triazine (TPTZ) and ascorbic acid were purchased from Sigma-Aldrich Co. (St. Louis, MO, USA).

### 2.2. Proximate Compositional Analysis

Proximate component analysis was performed according to the Korean Food Code method [20]. The moisture content was determined by atmospheric pressure drying at 105 °C, the ashing method was performed at 550 °C and the semi-micro Kjeldahl method and Soxhlet extraction method were performed to measure crude protein and crude lipid, respectively.

### 2.3. DPPH Radical Scavenging Activity

DPPH radical scavenging activity was measured by following the method proposed by Kim et al. [21]. Approximately 0.8 mL of 0.4 mM DPPH solution (dissolved in ethanol) was added to 0.2 mL of the diluted sample using distilled water (DW), and the reaction was performed at 23 °C for 10 min after vortexing. The absorbance was measured at 517 nm using a microplate reader (SpectraMax i3, Molecular Devices, Sunnyvale, CA, USA). The DPPH scavenging activity was calculated using Equation (1) (ascorbic acid was used as the positive control):

DPPH radical scavenging activity (%) = {1 − (A_experiment_/A_control_)} × 100 (1)

### 2.4. Ferric Reduction Antioxidative Power (FRAP) Assay

FRAP activity was measured as per the method proposed by Benzie et al. [22]. Sodium acetate (C_2_H_3_NaO_2_) and acetic acid (C_2_H_4_O_2_) were mixed to prepare sodium acetate buffer (pH 3.6). HCl (40 mM) and 2,4,6-Tripyridyl-s-triazine (TPTZ) were mixed to prepare a 10 mM TPTZ solution. The reaction solution was prepared by mixing sodium acetate buffer (pH 3.6), 10 mM TPTZ and 20 mM FeCl_3_·6H_2_O at a ratio of 10:1:1. Using 1.5 mL of the prepared solution, 150 μL of the diluted sample and 150 μL of the diluted DW were mixed and reacted for 4 min at 37 °C, and the absorbance was measured at 593 nm using a microplate reader.

### 2.5. Total Phenol and Total Flavonoid Content

#### 2.5.1. Total Phenol Content

The total phenol content was measured following the Folin–Ciocalteu method [23]. One milliliter of DW-diluted samples and 1 mL of 10% folin reagent were mixed. Then 1 mL of 2% Na_2_CO_3_ reagent was added, followed by mixing and incubation for 1 h in a dark place. The absorbance was measured at 750 nm. The total phenol content was determined from the standard curve prepared with gallic acid.

#### 2.5.2. Total Flavonoid Content

The total flavonoid content was determined with colorimetry following the method of Chun et al. [24]. To 0.5 mL of each sample, 1.5 mL of 95% EtOH, 0.1 mL of 10% aluminum nitrate, 0.1 mL of 1 M potassium acetate, and 2.8 mL of distilled water were added. The reaction was performed at 23 °C for 30 min and the absorbance was measured at 415 nm using a microplate reader. The flavonoid content was determined from the standard curve, prepared using rutin.

### 2.6. Analysis of Sinigrin Content

The pretreatments of the samples for the analysis of sinigrin in BJD and BJJ were carried out by modifying the method of Kim et al. [25]. First, 20 g of freeze-dried BJD and BJJ and 400 mL 80% ethanol was added, and the mixture was refluxed for 2 h at 70 °C, concentrated under reduced pressure and lyophilized. The instruments used for the analysis were a Waters 2695 Separation Module HPLC system and a Waters Photodiode Array Detector (Waters Co., Milford, MA, USA). Table 2 shows the conditions used for the analysis. The column used for the analysis was Sunfire (TM) C_18_ (4.6 mm × 250 mm, 5.0 μm, Waters Co., Milford, MA, USA).

### 2.7. Method Validation for Determination of Sinigrin

The validation method is based on the International Conference for Harmonization (ICH) guidelines [26], as are the specificity, accuracy, precision, linearity and detection limits of the method developed. The effectiveness of the assay was verified by using of limit of detection (LOD) and limit of quantitation (LOQ).

#### 2.7.1. Specificity

The specificity of the method was assessed by comparing the chromatograms and PDA spectra (UV) obtained from the standard sinigrin and the extract from *B. juncea*.

#### 2.7.2. Accuracy and Precision

Accuracy was verified through spike recovery tests. To perform this test, sample solutions were prepared by adding sinigrin at 3 different concentration levels (6.25, 25, 100 μg/mL) to *B. juncea* extracts with known concentrations. Accuracy is the amount recovered from the spikes compared to the known concentrations. Precision was evaluated by measuring the repeatability of the analysis in the intra-day and inter-day tests. Samples were injected three times and the results were expressed in relative standard measurement deviation (RSD%).

#### 2.7.3. Linearity

For the linearity study, a sinigrin standard was prepared at concentrations of 3.125, 6.25, 12.5, 25, 50, 100 and 200 µg/mL and measured repeatedly using HPLC, and a calibration curve indicating the relationship between the area and concentration ratio for the peak of the standard was prepared. Linearity was confirmed through a correlation coefficient (R^2^) value obtained from the prepared calibration curve.

#### 2.7.4. LOD and LOQ

LOD is defined as the lowest analyte concentration that the analytical process can reliably distinguish from the background level. It also defines the lowest concentration that can be quantified with as acceptable accuracy and precision as LOQ. The LOD and LOQ of the HPLC method were estimated from the signal-to-noise ratio (S/N). The LOD and LOQ for each analysis were calculated as the concentration levels at which the S/N reached 3 and 10, respectively.

### 2.8. Statistical Analysis

All the data are presented as mean ± SD (standard deviation) of samples in triplicate. The values were analyzed for significance by Student *t*-test or one-way ANOVA, followed by Duncan’s multiple range tests using SAS 9.4 (SAS Institute Inc., Cary, NC, USA). The differences were considered statistically significant if *p* < 0.05.

## 3. Results and Discussion

### 3.1. Comparison of Proximate Components

Table 3 shows the result of proximate compositional analysis of BJD and BJJ. The moisture content of BJD ranged from 91.96% to 93.49%, while that of the BJJ ranged from 92.36% to 93.03%. The crude ash content of BJD ranged from 1.47% to 1.55%, while that of BJJ ranged from 1.11% to 1.20%. The crude protein content of BJD ranged from 1.66% to 1.81%, while that of BJJ ranged 1.64% to 1.93%. The crude lipid content of BJD ranged from 0.42% to 0.59%, while that of BJJ ranged from 0.40% to 0.61%. In proximate component analysis, there was no significant difference between BJD and BJJ. However, the crude ash content of BJD was higher than that of BJJ.

### 3.2. DPPH Radical Scavenging Activity

**Figure 1:**
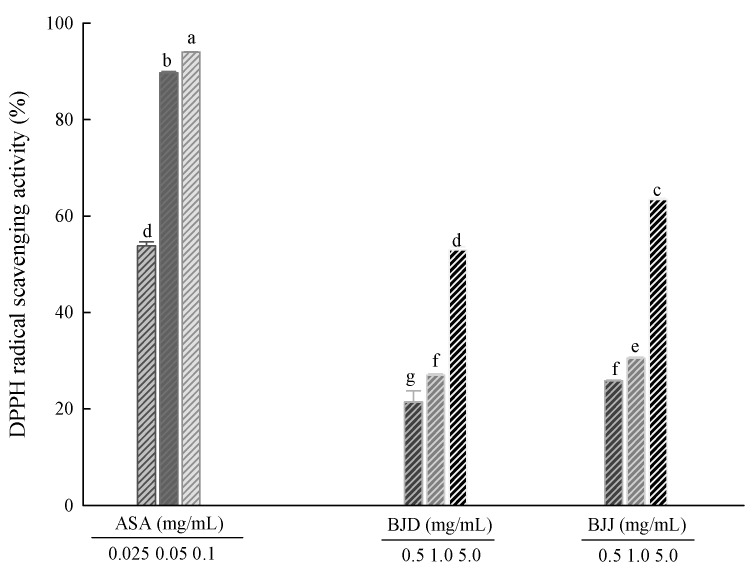
DPPH radical scavenging activity of BJJ and BJJ. Values are mean ± SD (*n* = 3). Means (bar value) not sharing a common letter (a–g) are significantly different (*p* < 0.05).

The DPPH radical scavenging activities of the BJD and BJJ extracts are shown in Figure 1. The DPPH radical scavenging activity of the BJD extracts was around 21.45% to 52.95% at 0.5, 1.0 and 5.0 mg/mL, and the DPPH radical scavenging activity of the BJJ extracts was around 25.84% to 63.41% at 0.5, 1.0 and 5.0 mg/mL. Thus, the DPPH radical scavenging activity was increased upon treatment with both concentrations of BJD and BJJ. Ascorbic acid, used as a positive control, showed high radical scavenging activity of 53.81% to 94.00% at concentrations of 0.025, 0.05 and 0.1 mg/mL, showing higher antioxidant activity than BJD and BJJ. DPPH is a very stable free radical and acts as a representative reactant in measuring antioxidant capacity. The DPPH radical scavenging method involves the discoloration of a purple color into yellow by free radical elimination through hydrogen donation in a phenol compound containing a hydroxyl radical or a substance having a flavonoid [27]. Thus, the BJJ extracts showed a better DPPH radical scavenging ability than the BJD extract by between 4.39% and 10.46% at each treatment concentration. Yun et al. [28] have comparatively analyzed the antioxidant activity of four sprout vegetables, such as green leaf mustard, BJD and cabbage among Brassicaceae species, and reported that BJD showed the highest antioxidant activity. This means that BJD has a high antioxidant activity among sprout vegetables. We confirmed that BJJ removes the DPPH radical more effectively and has higher antioxidant activity than BJD, having such high antioxidant activity.

### 3.3. FRAP Activity

**Figure 2:**
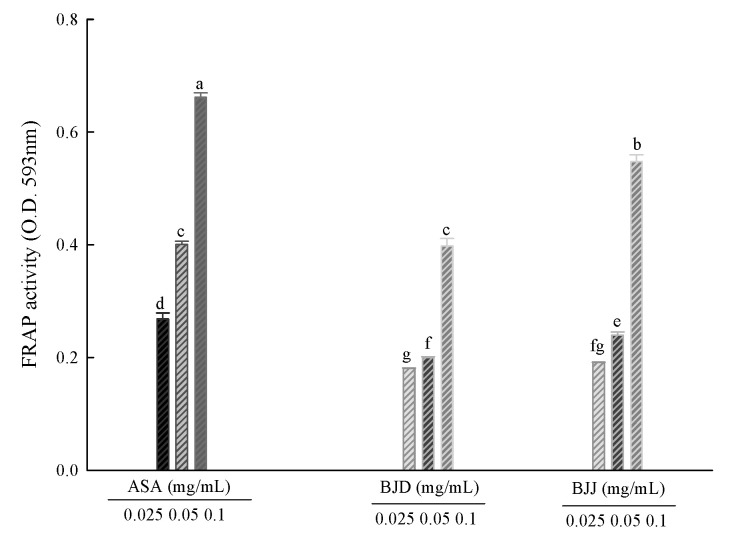
FRAP activity of BJD and BJJ. Values are mean ± SD (*n* = 3). Means (bar value) not sharing a common letter (a–g) are significantly different (*p* < 0.05).

The results of the FRAP assay are shown in Figure 2. The antioxidative activities of BJD extracts were 0.18 to 0.40 at 0.5, 1.0 and 5.0 mg/mL, and 0.19 to 0.55 at 0.5, 1.0 and 5.0 mg/mL for BJJ extracts. The BJJ extracts showed better FRAP activity than the BJD extracts. Ascorbic acid showed a high reduction capacity of 0.27 to 0.66 at concentrations of 0.025, 0.05 and 0.1 mg/mL, showing higher antioxidant activity than BJD and BJJ. The FRAP assay is a method for measuring antioxidant capacity by analyzing the conversion of the ferrous ion to ferric ions through the formation of the colored ferrous tripyridyl triazine complex. The ferric tripyridyl triazine (Fe^3+^-TPTZ) complex is reduced to ferrous tripyridyl triazine (Fe^2+^-TPTZ) by a reducing agent at low pH [29]. In the FRAP assay, the absorbance value itself indicates the reducing power of the sample, and the higher the antioxidant activity, the higher the absorbance value. As a result of FRAP activity, the absorbance of BJJ was 0.08 to 0.11 higher than that of the BJD extract. This suggests that BJJ has better reducing power and antioxidant ability.

### 3.4. Total Phenol and Total Flavonoid Content

Table 4 shows the total phenol and total flavonoid content of BJJ and BJD. The total phenol content of BJD ranged from 5.73 to 7.15 mg gallic acid equivalent (GAE)/g, and for BJJ it ranged from 9.61 to 9.93 mg GAE/g. The total flavonoid content of BJD ranged from 20.35 to 21.17 mg rutin equivalent (RE)/g and of BJJ ranged from 34.41 to 35.09 mg RE/g. BJJ contained approximately 3.24 mg GAE/g more polyphenols than BJD, and that of BJJ contained approximately 13.89 mg RE/g more flavonoid content than BJD. The differences were statistically significant. The phenolic compounds that are present in the plants are reported to have high antioxidant functions. As the total polyphenol content increases, the physiological activity, such as antioxidation, is increased [30,31]. Therefore, the antioxidant capacity of BJJ, due to phenolic compounds, is expected to show better activity than BJD. This may explain why BJJ showed better antioxidant activity than BJD in the DPPH and FRAP assays.

### 3.5. Method Validation

#### 3.5.1. Specificity

**Figure 3:**
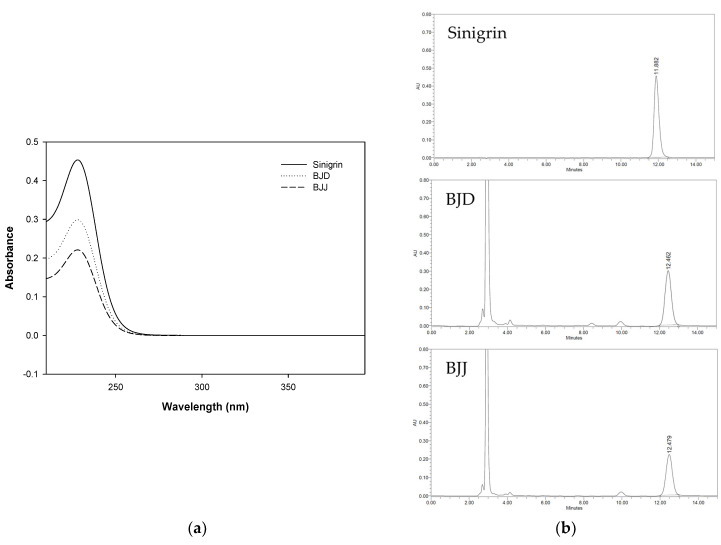
(**a**) PDA spectrums and (**b**) HPLC chromatogram of sinigrin, BJD and BJJ.

As a result of confirming the sinigrin peak by comparing the chromatogram of the standard solution and the *B. juncea* extract, it was confirmed that it was selectively separated without interference from other components, as shown in Figure 3. In addition, when analyzing the *B. juncea* extract, the retention time of the standard solution and the retention time of the two substances of the extract were identical, and the PDA spectrum also showed the same spectrum, thereby confirming the specificity of this test method.

#### 3.5.2. Accuracy and Precision, Linearity, LOD and LOQ

Accuracy was indicated by measuring intra-day and inter-day recovery rates. As shown in Table 4, the intra-day accuracy of sinigrin was 97.12% to 101.01%, and inter-day accuracy was 98.65% to 101.62%, showing excellent accuracy. The precision of the method was assessed by intra-day and inter-day variations. The method showed good precision, with intra-day and inter-day variations of 0.08% to 0.73% (RSD%) and 0.07% to 0.66%, respectively, as shown in Table 5. The calibration curve showed excellent linearity (R^2^ = 1). The LOD of sinigrin was measured to be 0.08 μg/mL, and the LOQ was 0.24 μg/mL, as shown in Table 6. From the above results, it was found that sinigrin in *B. juncea* can be measured using HPLC and quantitative analysis. Based on these results, a simple and reliable HPLC method with PDA detector for the quantification of sinigrin was developed and validated for its specificity, linearity, precision, accuracy, LOD and LOQ.

### 3.6. Analysis of Sinigrin Contents

The standard curve for the quantitative analysis was prepared at the concentrations of 3.125, 6.25, 12.5, 25, 50, 100 and 200 μg/mL sinigrin. BJD and BJJ were prepared at a concentration of 10 mg/mL and used for analysis. The results of the comparative analysis of the sinigrin components contributing to the unique fragrance of *B. juncea* are shown in Figure 3 and Table 7. The sinigrin contents of BJD ranged from 15.89 to 16.35 mg/g and that of BJJ ranged from 11.56 to 11.92 mg/g. BJD contained about 4.43 mg/g more sinigrin than BJJ. Sinigrin and their degradation products are known to have a characteristic bitter taste and aroma in vegetables [32]. Thus, polyphenolic compounds and antioxidant capacity are better in BJJ than BJD, but the peculiar aroma of *B. juncea* is thought to be better in BJD than BJJ.

## 4. Conclusions

This study was carried out to provide basic data on the *B. juncea* cultivated in the Jeongseon province of Gangwon—done by comparing the components of BJD and BJJ. The measurement of DPPH radical scavenging activity and FRAP activity confirmed that BJD and BJJ extracts showed increased activity, depending on the concentration, and thus the antioxidant capacity was improved. The total phenol content was measured as 6.56 ± 0.60 mg GAE/g and 9.80 ± 0.14 mg GAE/g in the BJD and BJJ extracts, respectively. BJJ was found to contain approximately 3.24 mg GAE/g more than BJD. The total flavonoid content was found to be 20.92 ± 0.41 mg RE/g and 34.81 ± 0.29 mg RE/g in the BJD and BJJ extracts, respectively. BJJ was found to contain approximately 13.89 mg RE/g more flavonoid content than BJD. Based on these results, the food components and antioxidant activities of BJD and BJJ were examined. Analysis of sinigrin content, which affects the spicy taste and flavor of *B. juncea*, revealed a content of 16.16 ± 0.24 mg/g in BJD and 11.73 ± 0.18 mg/g in BJJ. By comparing the antioxidant activity and analyzing the chemical components that can contribute to physiological activity, it was confirmed that the antioxidant activity of BJJ was better than that of BJD, and the possibility and necessity of future research on BJJ as a dietary supplement was confirmed.

## Figures and Tables

**Table 1:** Environmental conditions ^1^ for the *Brassica juncea* used in the study.

Location	Rainfall (mm)	Temperature (°C)	Humidity (%)
Dolsan, Yeosu city	148.3	18.1	70
Jeongseon city	28.0	13.1	73

^1^ October, 2017.

**Table 2:** HPLC condition for analysis of sinigrin.

Instrument	Conditions
Column	Sunfire™ C_18_, (5.0 μm, 4.6 mm × 250 mm)
Column temp.	30 °C
Mobile phase (isocratic)	Isocratic HPLC water containing 0.5 M ammonium sulfate
Detector	PDA detector (228 nm)
Flow rate	1.0 mL/min
Injection volume	40 μL
Run time	15 min

**Table 3:** Comparison of proximate composition of BJD and BJJ.

Composition (%)	BJD		BJJ	
	Mean ± SD	RSD ^1^	Mean ± SD	RSD
Moisture	92.72 ± 0.63	0.68	92.66 ± 0.28	0.3
Crude ash	1.52 ± 0.04 *	2.42	1.15 ± 0.04	3.45
Crude protein	1.74 ± 0.07	4.45	1.83 ± 0.01	7.24
Crude lipid	0.53 ± 0.07	14.92	0.52 ± 0.08	16.93

^1^ RSD: Relative standard deviation; * Values are mean ± SD (*n* = 3). Data were statistically analyzed by independent sample *t*-test. Values are significantly different between BJD and BJJ (*p* < 0.05).

**Table 4:** Total phenol and total flavonoid contents of BJD and BJJ.

Sample	Total Phenol Content (mg GAE ^1^/g)		Total Flavonoid Content (mg RE ^2^/g)	
	Mean ± SD	RSD ^3^	Mean ± SD	RSD
BJD	6.56 ± 0.60	9.23	20.92 ± 0.41	1.95
BJJ	9.80 ± 0.14 *	1.43	34.81 ± 0.29 *	0.85

^1^ GAE: Gallic acid equivalent; ^2^ RE: Rutin equivalent; ^3^ RSD: Relative standard deviation; * Values are mean ± SD (*n* = 3). Data were statistically analyzed by independent sample *t*-test. Values are significantly different between BJD and BJJ (*p* < 0.05).

**Table 5:** Precision and accuracy of sinigrin analysis.

Analytes		Concentration (μg/mL)	Mean ± SD (μg/mL)	RSD ^1^ (%)	Recovery (%)
Sinigrin	Intra-day ^2^	6.25	6.07 ± 0.03 *	0.55	97.12 ± 0.44
		25	25.25 ± 0.18	0.73	101.01 ± 0.60
		100	99.65 ± 0.08	0.08	99.65 ± 0.07
	Inter-day ^3^	6.25	6.17 ± 0.02	0.39	98.65 ± 0.39
		25	25.41 ± 0.17	0.66	101.62 ± 0.67
		100	99.76 ± 0.07	0.07	99.76 ± 0.07

^1^ RSD: Relative standard deviation; ^2^ Intra-day: Three times per day; ^3^ Inter-day: One analysis per day for three days; * Values are mean ± SD (*n* = 3).

**Table 6:** Correlation coefficient of the calibration curves, LOD ^1^ and LOQ ^2^ of sinigrin analysis.

Analyte	Range (μg/mL)	Slope	Intercept	Correlation Coefficient (R^2^)	LOD (μg/mL)	LOQ (μg/mL)
Sinigrin	3.125~200	41,679	1153.55	1	0.08	0.24

^1^ LOD: Limit of detection; ^2^ LOQ: Limit of quantitation.

**Table 7:** The sinigrin content of BJD and BJJ.

Analytes	BJD		BJJ	
	Mean ± SD	RSD ^1^ (%)	Mean ± SD	RSD (%)
Sinigrin (mg/dry weight g)	16.16 ± 0.24 *	1.48	11.73 ± 0.18	1.57

^1^ RSD: Relative standard deviation; * Values are mean ± SD (*n* = 3). Data were statistically analyzed by independent sample *t*-test. Values are significantly different between BJD and BJJ (*p* < 0.05).
